# Overexpressed p-S6 associates with lymph node metastasis and predicts poor prognosis in non-small cell lung cancer

**DOI:** 10.1186/s12885-022-09664-4

**Published:** 2022-05-20

**Authors:** Yaoxiang Tang, Jiadi Luo, Ying Zhou, Hongjing Zang, Yang Yang, Sile Liu, Hongmei Zheng, Jian Ma, Songqing Fan, Qiuyuan Wen

**Affiliations:** 1 Department of Pathology, The Second Xiangya Hospital, Central South University, Changsha, 410011 Hunan China; 2 Cancer Research Institute of Central South University, Hunan Key Laboratory of Nonresolving Inflammation and Cancer, Central South University, Changsha, 410011 Hunan China

**Keywords:** P-S6, Lymph node metastasis, Prognosis, Non-small cell lung cancer

## Abstract

**Background:**

Ribosomal protein S6 (S6), a downstream effect media of the AKT/mTOR pathway, not only is a part of 40S small subunit of eukaryotic ribosome, but also involves in protein synthesis and cell proliferation during cancer development.

**Methods:**

In present study, we explore the association between phosphorylated S6 (p-S6) protein expression and clinicopathological features as well as prognostic implications in NSCLC. P-S6 was detected in tissue microarrays (TMAs) containing 350 NSCLC, 53 non-cancerous lung tissues (Non-CLT), and 88 cases of matched metastatic lymph node lesions via immunohistochemistry (IHC). Transwell assays and wound healing assay were used to assess the effects of p-S6 inhibition on NSCLC cell metastasis.

**Results:**

The p-S6 expression in NSCLC was more evident than that in Non-CLT (*p <* 0.05). Compared to NSCLC patients who have no lymph node metastasis (LNM), those with LNM had higher p-S6 expression (*p =* 0.001). Regardless of lung squamous cell carcinoma (SCC) or adenocarcinoma (ADC), p-S6 was increased obviously in metastatic lymph nodes compared with matched primary cancers (*p =* 0.001, *p =* 0.022, respectively). Inhibition of p-S6 decreased the metastasis ability of NSCLC cells. In addition, p-S6 was an independent predicted marker for LNM in patients with NSCLC (*p <* 0.001). According to survival analysis, patients with highly expressed p-S6 had a lower survival rate compared with that with lower expression (*p =* 0.013). P-S6 is an unfavorable independent prognostic factor for NSCLC patients (*p =* 0.011).

**Conclusion:**

Increased expression of p-S6 is not only a novel predictive biomarker of LNM but also poor prognosis in NSCLC.

**Supplementary Information:**

The online version contains supplementary material available at 10.1186/s12885-022-09664-4.

## Background

Lung cancer is a clinical malignant tumor with highest incidence, with about 2 million new lung cancer cases annually, and its mortality rate is also the highest worldwide [1]. 80 to 90% of lung cancer are diagnosed as non-small cell lung cancer (NSCLC), which is mainly classified into adenocarcinoma (ADC) and squamous cell carcinoma (SCC) [2]. Lung cancer patients’ survival varies depending on lymph node metastasis (LNM), clinical stage, geographic region, and other factors. The 5-year survival of NSCLC patients in clinical stageIis about 57%, which is far more than that of patients with stage IV (that is less than 5%) [3]. Unfortunately, more than half of the NSCLC patients are diagnosed in advanced clinical stages, often accompanied by LNM and distant metastasis, which are important reasons for the poor prognosis [3, 4]. Therefore, it is an urgent need to find new biomarker for early detection of LNM of NSCLC and predict the prognosis.

Recently, the protein kinase B (AKT)/mammalian target of rapamycin (mTOR) signal pathway is over-activated in numerous tumors, including NSCLC, and associated with tumor angiogenesis, invasion, metastasis and so on [5–7]. Ribosomal protein S6 (S6), a part of 40S small subunit of eukaryotic ribosome, is a famous downstream effect media of the AKT/mTOR pathway [8, 9]. As the first ribosomal protein proved to undergo inducible phosphorylation, S6 is mostly induced by activated 70 kDa S6 Kinases (S6K) at five phosphorylation sites (Ser235, Ser236, Ser240, Ser244, Ser247), mainly Ser236 [9–12]. Phosphorylated S6 (p-S6) plays a crucial part in protein synthesis, cell size control and cell proliferation as increasing the affinity of the ribosome and mRNAs and improving the efficiency of protein translation [13]. In addition, p-S6 is also the key effector of mTOR in regulating cell size, whose decreased expression results in smaller cell size and reflects the growth defect [14, 15]. Overexpression of p-S6 is found in various solid tumors, such as gastric cancer, glioblastomas, and renal cell carcinomas (RCCs), and associated with poor prognosis [16–18]. S6 phosphorylation was even associated with malignant potential and glucose metabolism of intraductal papillary mucinous neoplasm of the pancreas [19]. In addition, the phosphorylation of S6 is considered to contribute to acquired resistance to MAPK pathway inhibitors in cancers, suggesting that p-S6 plays an essential role in the mechanism of anti-cancer drugs [20, 21].

However, the prognostic implications of p-S6 are poorly understood in NSCLC. In this present research, we have measured the level of p-S6 expression in tissue microarrays (TMAs) of non-cancerous lung tissues (Non-CLT) and NSCLC by immunohistochemistry (IHC) and decreased p-S6 expression in NSCLC cell lines with mTOR inhibitor (RAD001), to estimate not only the association between p-S6 and clinicopathological/prognostic features of NSCLC patients, but also the alteration of NSCLC cell migration and invasion ability.

## Material and Methods

### Patients and tissue samples

All tissue specimens which were collected by surgical resection acquired from the Thoracic Surgery Department of the Second Xiangya Hospital of Central South University (CSU) between 2003 and 2013. Approval of all protocols were obtained from the Institutional Human Experiment and Ethics Committee of the Second Xiangya Hospital of CSU (Changsha, China) (approval No. S039/2011), and approval of all protocols were obtained. 350 cases of NSCLC, 53 cases of Non-CLT, and 88 cases of matched metastatic lymph node lesions were included in this research. Patients in the study had definite histological diagnosis on the basis of 2015 WHO Classification of lung cancer [22], and were comprehensively staged as per the Eighth Edition Lung Cancer Stage Classification [23]. Complete clinicopathological data obtained from medical records were available for all patients (Supplementary Table).

### Cells lines and reagents

Two human NSCLC cell lines, A549 and SPC-A1, were from the Cell Bank, Chinese Academy of Sciences (Shanghai, China) and those were grown in 37 °C, 5% CO_2_ incubator. Both cell lines were culture in RPMI-1640 (BI, Israel) medium comprising 10% fetal bovine serum (BI, Israel). The mTOR inhibitor Everolimus (RAD001) was procured from Selleckchem, USA. Rabbit antibodies against p-S6 (S235/236) (#4857) were obtained from Cell Signaling Technology, USA. Mouse antibodies against S6 (cat no. 66886-1-Ig) and β-Actin (cat no. 66009-1-Ig) were purchased from proteintech, USA. Peroxidase-conjugated Affinipure Goat anti-Rabbit IgG (cat no. HG-SAR00002a) and anti-Mouse IgG (cat no. YG-SAM00001a) were purchased from HonorGene, China.

### Construction of the tissue microarrays

As previously described, tissue microarrays (TMAs) were constructed using paraffin-embedded tissue of NSCLC and Non-CLT [24]. The perforation diameter of each sample was 0.6 mm. For TMAs of NSCLC, each case included two tumor cores, of which 88 cases each also contained two matched metastatic lymph node cores. For TMAs of Non-CLT, two cores per case was included. The average score of the two cores was regarded as the final score for each case.

### Immunohistochemistry and scores

IHC for p-S6 in TMAs was carried out and the conditions of antibody staining were adjusted as described previously [25]. Briefly, primary antibody for p-S6 (1:800 of Ser235/236) was applied to detect its expression level. The positive control slide and negative control slide were included in each experiment. The IgG isotype-matched antibody was applied as negative contrast to confirm the antibody specificity.

Immunoreactivity was assessed semi-quantitatively by two independent pathologists. The staining intensity score was multiplied by percentage score of positive expressed tumor cells as the total score. Specifically, staining intensity was scored as 0 to 3 (0 for negative; 1 for mild expression; 2 for moderate expression; 3 for strong expression). The percentage score was identified as 0 to 4 in view of positive cytoplasmic staining: 0 = 0%; 1 = 1-25%, 2 = 26-50%, 3 = 51-75%, 4 = 76-100%. The range of total score was from 0 to 7. Cutoff level which determined according to overall survival (OS) of NSCLC patients was 2. Expression of p-S6 in tumor cells of which total score is more than 2 was considered high expression and others were regarded as low expression. Agreement between the two assessors is 96%, borderline and ambiguous cases are resolved through discussion.

### Western blotting

As previously described [5, 26], SPC-A1 and A549 cells were seeded and then treated with DMSO or 5 nmol/L RAD001 for 24 h, preparation of protein lysates and western blotting analysis were performed. The blots were cut prior to hybridisation with antibodies. The antibodies were used as follows: 1:1000 of Ser235/236 for anti-p-S6 and 1:5000 for anti-β-Actin.

### Wound healing assay

1*10^5^ SPC-A1 and A549 cells were seeded and then treated with DMSO or 5 nmol/L RAD001 at about 60% confluence and cultured until 90%. Serum free RPMI-1640 was used to replace the culture medium and wounds were scratched with the tip of 10 μl pipette in each well. The size of wounds was observed using inverted microscope (Leica, Germany) and images were captured at 0 h, 24 h and 48 h. The data presented were repeated in triplicate. Wound healing percentage (%) = (1 – scratch area at t / scratch area at 0 h) * 100% (t means 24 h or 48 h).

### Transwell assays

24-well chambers (Costar 3422; Corning, USA) were used to perform transwell migration assays. A549 and SPC-A1 cells were treated with DMSO or 5 nmol/L RAD001 for 24 h at 37 °C. Then 5*10^4^ cells were extracted from each group and suspended in 200 μl of serum-free RPMI-1640 to the upper chamber, while 10% FBS RPMI-1640 was added to the lower room. The cells could migrate through the membrane for 12 h to 48 h. The membrane was fixed for 20 min in 4% paraformaldehyde solution and dyed with crystal violet for 20 min at room temperature. The count of cells under the cell membrane was calculated. The implementation of invasion assay protocol be analogous to that of migration assay, except that Matrigel was coated on the upper cavity and the incubation time was 1 h (356,234, Corning, USA).

### Statistical analysis

The difference expression pattern of p-S6 between Non-CLT and NSCLC, as well as the association between p-S6 and clinicopathological features were analyzed by Chi-square (χ^2^) test. The difference between the metastatic lymph nodes and matched primary tumors was also analyzed by Chi-square(χ^2^) test. Multi-logistic regression method was performed to identify whether p-S6 is an independent marker for LNM of NSCLC. Survival rate curve was evaluated by Kaplan-Meier analysis, and comparisons were analyzed via log-rank test. Furthermore, cox proportional hazards model was performed for determining independent prognostic markers. The deviation between control and RAD001 groups was analyzed using one way t-test. All these statistical analyses were put into effect with SPSS Statistics software (version 24). Numerical data were presented as mean ± SEM. *P* < 0.05 (Two-sided) indicates that the result is statistically significant.

## Results

### P-S6 was significantly overexpressed in NSCLC

**Fig. 1:**
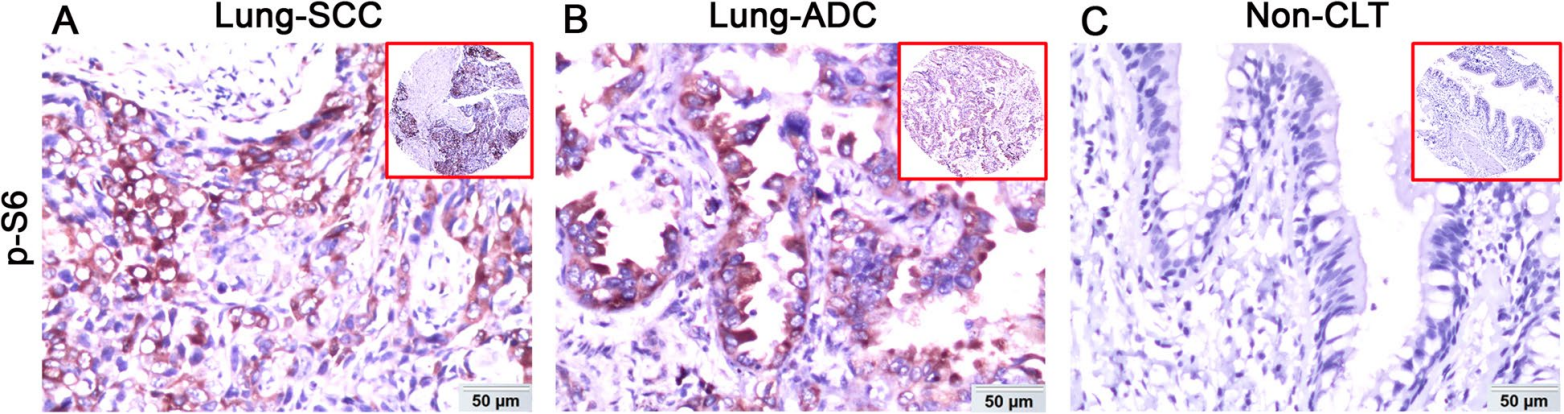
Evaluation of p-S6 expression in lung SCC, lung ADC and Non-CLT. P-S6 was strongly positive in cell cytoplasm in lung SCC (**A**) and lung ADC (**B**). In Non-CLT (**C**), p-S6 staining was negative. (200×, IHC, DAB staining)

Firstly, we detected the intracellular localization and the level of p-S6 expression by IHC in a total of 403 samples, of which 350 were NSCLC. The results indicated that p-S6 was mainly observed in the cell cytoplasm of lung SCC, ADC and Non-CLT (Fig. 1). Quantification of p-S6 expression showed that its percentage of high expression was 30.5% (47/154) in lung SCC, 62.2% (122/196) in lung ADC and 15.1% (8/53) in Non-CLT, respectively. As shown in Supplementary Fig. 1, compared with Non-CLT, the level of p-S6 protein was obviously increased both in lung SCC and ADC (*p =* 0.031, *p <* 0.001, respectively).

### Association between p-S6 and clinicopathological features

As data showed in Table 1, p-S6 in female was higher than that in male (*p <* 0.001). Patients with lung ADC had obviously higher p-S6 expression level than patients with SCC (*p <* 0.001). Overexpressed p-S6 was significantly correlated with LNM (*p =* 0.001). Moreover, patients with high p-S6 expression level suffered a lower overall survival (OS) rate than that with low expression (*p =* 0.005). However, there was not significantly association between p-S6 expression and other features, such as clinical stages, pathological grade and age (all *p* > 0.05).

**Table 1:** Analysis of the association between the expression level of p-S6 and clinicopathological features of NSCLC (*n =* 350)

Clinicopathological features (n)	p-S6			
	N	High (%)	Low (%)	*P-*value
**Age (years)**				
≤ 50	95	48 (50.5)	47 (49.5)	
>50	255	121 (47.5)	134 (52.5)	0.632
**Gender**				
Male	266	114 (42.9)	152 (57.1)	
Female	84	55 (65.5)	29 (34.5)	0.000^a^
**Clinical stage**				
Stage _I-II_	151	75 (49.7)	76 (50.3)	
Stage _III_	199	93 (46.7)	106 (53.3)	0.519
**LN status**				
LNM	210	117 (55.7)	93 (44.3)	
No LNM	140	52 (37.1)	88 (62.9)	0.001^a^
**Histological type**				
SCC	154	47 (30.5)	107 (69.5)	
ADC	196	122 (62.2)	74 (37.8)	0.000^a^
**Pathological grade**				
Well/ Moderate	152	77 (50.7)	75 (49.3)	
Poor	198	92 (46.5)	106 (53.5)	0.452
**Survival status**				
Alive	187	77 (41.2)	110 (58.8)	
Dead	163	92 (56.4)	71 (43.6)	0.005^a^

^a^Chi-square test, statistically significant difference (*P <* 0.05). Abbreviations: *ADC* Adenocarcinoma, *SCC* squamous cell carcinoma, *LNM* Lymph node metastasis

### Impact of p-S6 expression on LNM in NSCLC

**Fig. 2:**
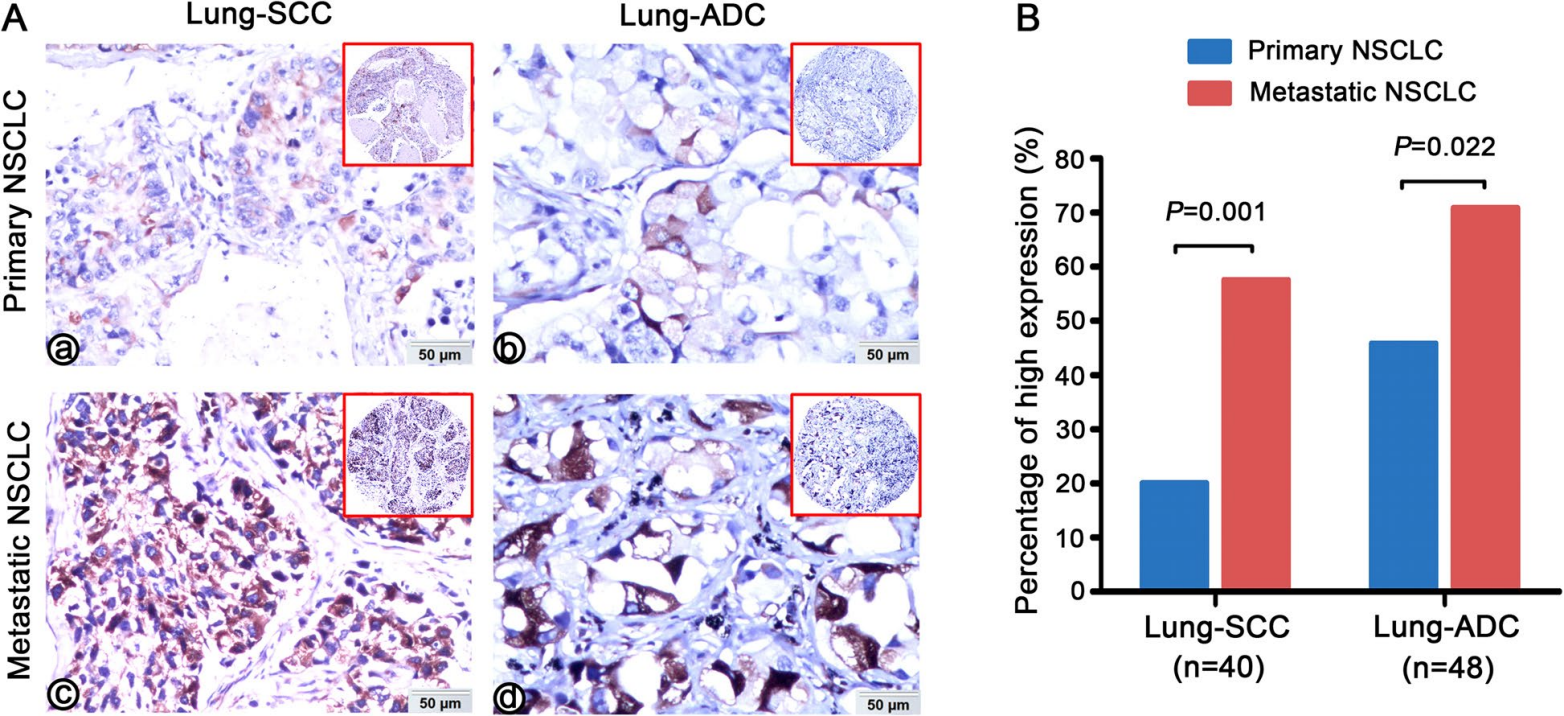
Comparison of p-S6 expression in metastatic NSCLC and matched primary cancer. **A** P-S6 was strongly positive expressed in cell cytoplasm in primary lung SCC (A-a), primary lung ADC (A-b) and mild positive staining in its matched LNM lesions (A-c, A-d). **B** Whether it is lung SCC or lung ADC, the expression level of p-S6 in metastatic cancer foci is significantly higher than that in primary cancer foci (*p =* 0.001, *p =* 0.022, respectively)

Among the 350 NSCLC patients, 210 cases had LNM and 140 cases were free from LNM. NSCLC patients with LNM had obviously higher p-S6 expression compared with those without LNM (*p =* 0.001, Table 1). We also evaluated the level of p-S6 in primary NSCLC tissues and its matched LNM lesions. As shown in Fig. 2, regardless of lung SCC or ADC, the percentage of high p-S6 expression is significantly higher in metastatic lymph node lesions with compared with the matched primary cancer (*p =* 0.001, *p =* 0.022, respectively). To determine whether p-S6 expression was the independent predicted parameter for LNM in NSCLC, a multivariate logistic regression analysis was carried out. As mentioned in Table 2, highly expressed p-S6 (*p =* 0.001), clinical stages (*p <* 0.001), and age (*p =* 0.010) were notably correlated with LNM status of NSCLC patients. These results indicated that overexpressed p-S6 is an independent factor for LNM of NSCLC regardless of other parameters.

**Table 2:** Multivariate logistic analysis of LNM factors in NSCLC patients

Variables						95.0% CI for Exp (B)	
	B	S.E.	Wald	P-vaiue	Exp(B)	Lower	Upper
**p-S6 expression**	0.921	0.270	11.644	0.001*	2.512	1.480	4.262
**Clinical stages**	1.957	0.264	54.968	0.000*	7.068	4.220	11.880
**Histological type**	−0.026	0.276	0.009	0.925	0.974	0.567	1.674
**Pathological grade**	−0.051	0.256	0.039	0.843	0.951	0.575	1.571
**Age**	−0.759	0.293	6.688	0.010*	0.468	0.263	0.832
**Gender**	0.607	0.324	3.516	0.061	1.835	0.973	3.462

Abbreviations: *S. E* Standard error, *Exp(B)* Odds ratio, *CI* Confidence interval, *LNM* Lymph node metastasis. * *P* < 0.05

### Inhibition of p-S6 reduced the cell migration and invasion of NSCLC

**Fig. 3:**
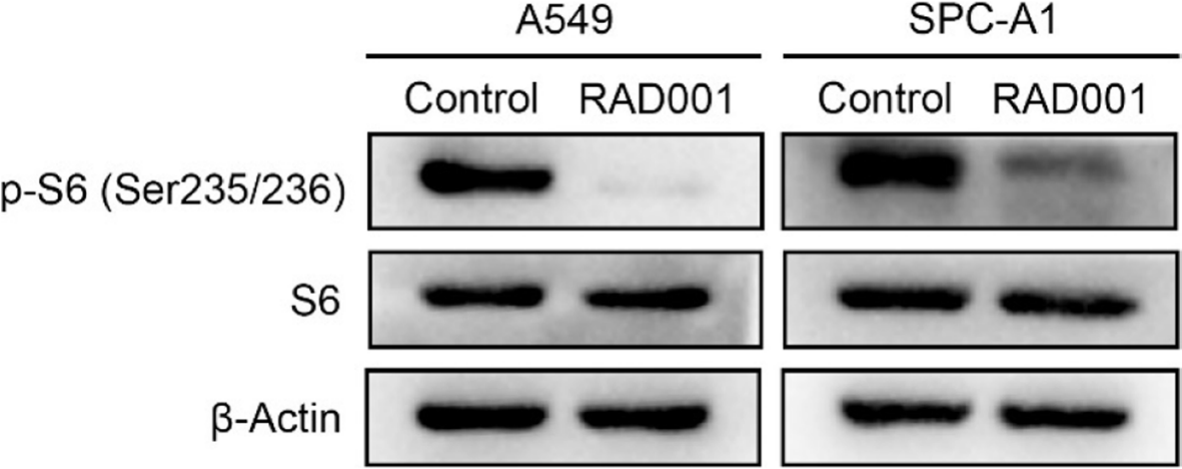
Effect of RAD001 on expression of p-S6 protein in NSCLC cell lines (A549 and SPC-A1). NSCLC Cells were treated with DMSO or RAD001 (5 nmol/L) for 24 h. In A549 and SPC-A1, compared with Control group, p-S6 protein in RAD001 groups were significantly decreased. Original blots are presented in Supplementary Fig. 2

**Fig. 4:**
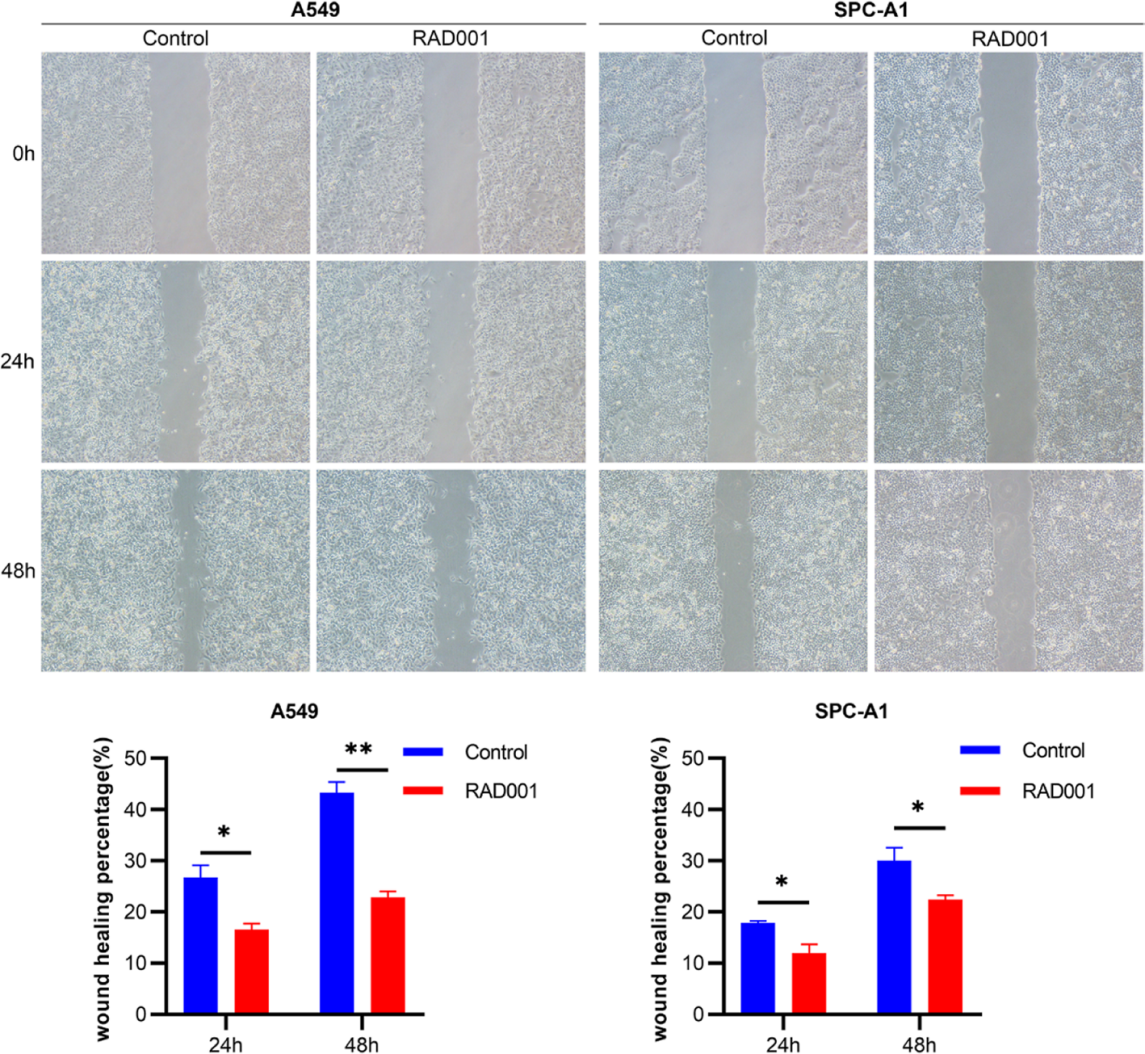
p-S6 inhibition reduced wound healing of NSCLC cell lines (A549 and SPC-A1). Low expression of p-S6 decreased the wound healing percentage of A549 and SPC-A1. *p < 0.05 and **p < 0.01, Control vs. RAD001. (200×)

**Fig. 5:**
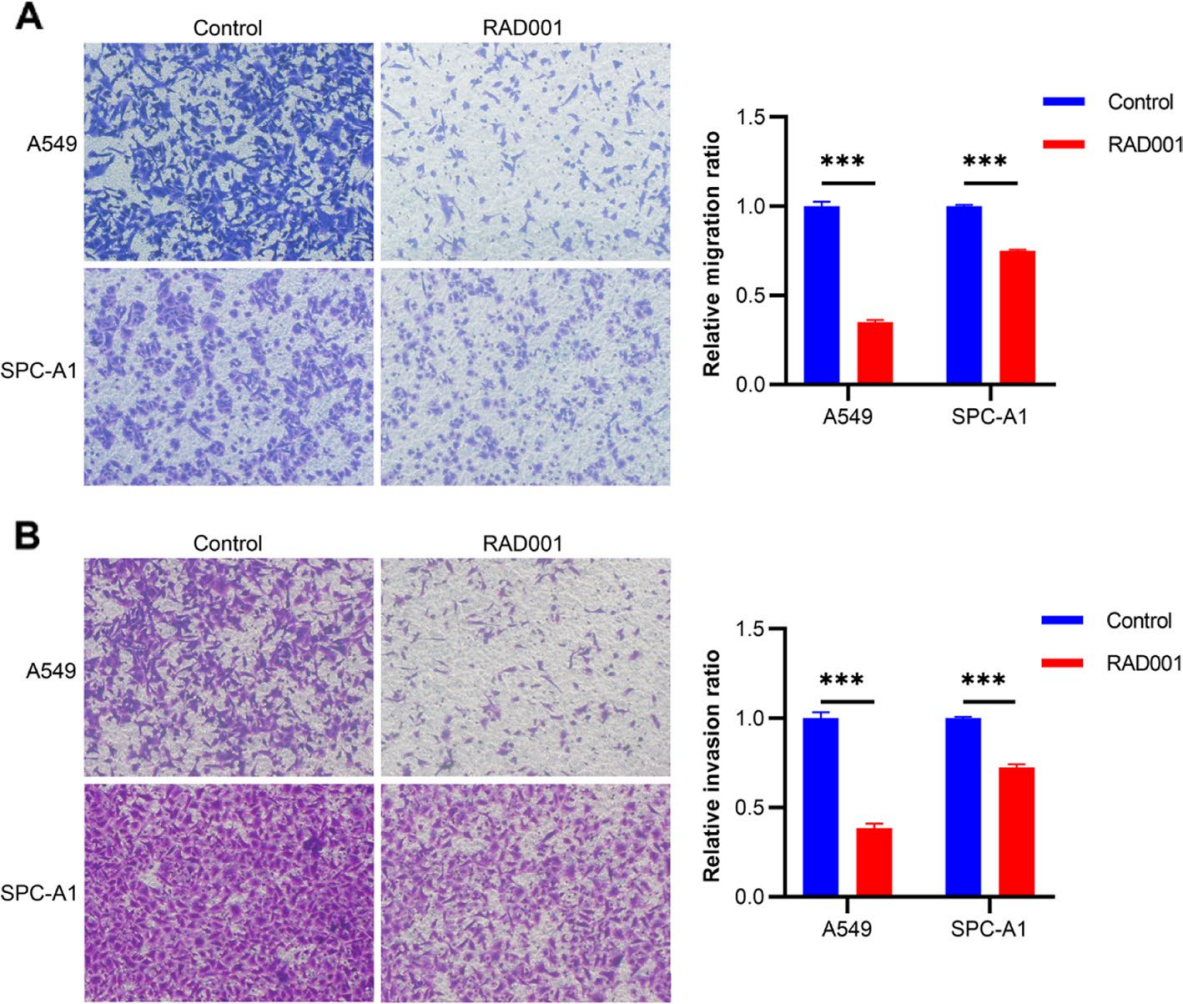
Effects of p-S6 inhibition on migration and invasion of NSCLC cell lines (A549 and SPC-A1). A549 and SPC-A1 cells were treated with 5 nmol/L RAD001 and were subjected to transwell assay to detect cell migration and invasion activity. **A** Results showed that migration of cell lines, A549 and SPC-A1, decreased following treatment with RAD001. **B** Inhibition of p-S6 decreased the invasion ability of cell lines A549 and SPC-A1. ***p < 0.001, Control vs. RAD001. (200×)

The alteration of cell migration and invasion ability of NSCLC were assessed after inhibition of p-S6 expression level in NSCLC cell lines (A549 and SPC-A1). Firstly, we evaluated the inhibition efficiency by Western blotting after the cell lines were treated with RAD001. According to our previous research [5, 26], NSCLC cells was treated with RAD001 at 5 nmol/L for 24 h. Figure 3 showed that p-S6 expression was significantly reduced after treatment with RAD001. Secondly, to detect the effect of p-S6 on the cell migration, wound healing and transwell migration experiments were conducted. Figure 4 showed that compared with the control group, the wound healing percentage of RAD001 group was decreased by 10.19 and 20.44% at 24 h and 48 h in A549 cells (*p =* 0.018, *p =* 0.001, respectively), by 5.90 and 7.55% in SPC-A1 cells (*p =* 0.028, *p =* 0.048, respectively), respectively. Figure 5A revealed that the inhibition of p-S6 expression in A549 and SPC-A1 cell lines decreased the migration ability by transwell migration assay (both *p <* 0.001). Moreover, the cell invasion assay demonstrated that p-S6 inhibition in NSCLC cell lines reduced the number of invaded cells (both *p <* 0.001) (Fig. 5 B). Together, these results revealed that inhibition of p-S6 expression could reduce the cell migration and invasion of NSCLC.

### Impact of p-S6 expression on patients’ prognosis

**Fig. 6:**
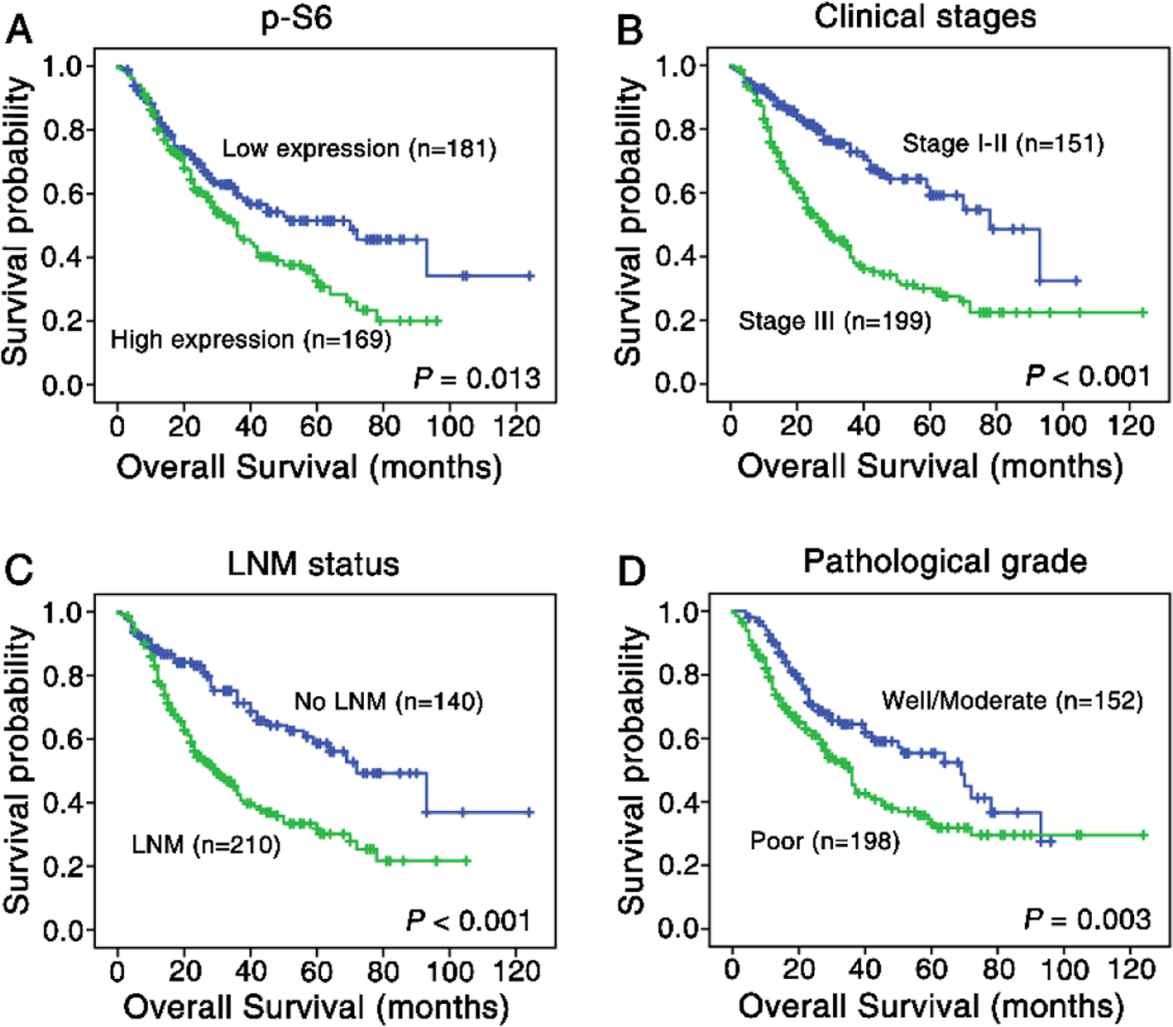
Kaplan-Meier survival estimates in patients with lung SCC and ADC. **A** NSCLC patients with highly expressed p-S6 protein showed worse OS rate in comparison to patients with low p-S6 expression level (*p =* 0.013). **B** Compared to NSCLC patients in stageI-II, patients with stage III had worse OS rates (*p <* 0.001). **C** NSCLC patients with LNM owned poor prognosis compared with patients without LNM (*p <* 0.001). **D** NSCLC patients whose differentiation was poor had lower OS rate than those with well and moderate differentiation (*p =* 0.003)

In univariate survival analysis, survival rate was estimated by Kaplan-Meier method in 350 NSCLC patients, and comparison was performed via log-rank test. NSCLC patients with highly expressed p-S6 had a poor prognosis compared to those with lowly expressed p-S6 (*p =* 0.013) (Fig. 6 A & Table 3). The OS rate of NSCLC patients with advanced clinical stage (stage III) was lower relative to those with early stages (stageI-II) (*p <* 0.001) (Fig. 6 B & Table 3). Of note, patients without LNM have higher OS than those with LNM (*p <* 0.001) (Fig. 6 C & Table 3). Meanwhile, compared with NSCLC patients with well and moderated pathological grade, lower OS could be seen in those with poor differentiation (*p =* 0.003) (Fig. 6 D & Table 3).

**Table 3:** Summary of univariate/multivariate analysis for overall survival in patients with NSCLC (*n =* 350)

Variables	Univariate analysis			Multivariate analysis		
	Average survival time (SE)	95%CI	*P*-value	Exp (B)	95.0%CI	*P*-value
**p-S6**						
High expression	44.561 (3.050)	38.583-50.540	0.013*	1.549	1.105-2.173	0.011*
Low expression	67.500 (5.336)	57.041-77.958				
**Clinical stages**						
Stage _I-II_	68.200 (4.195)	59.977-76.423	0.000*	1.980	1.356-2.893	0.000*
Stage _III_	47.862 (3.834)	40.347-55.378				
**LN status**						
LNM	44.969 (3.201)	38.695-51.243	0.000*	1.609	1.098-2.360	0.015*
No LNM	74.932 (5.919)	63.330-86.533				
**Histological type**						
SCC	63.464 (5.825)	52.046-74.882	0.240	1.137	0.806-1.603	0.464
ADC	50.588 (3.367)	43.988-57.188				
**Pathological grade**						
Well/moderated	58.662 (3.509)	51.784-65.541	0.003*	1.517	1.094-2.104	0.013*
Poor	54.226 (4.164)	46.066-62.387				
**Age**						
≤ 50	45.395 (3.633)	38.274-52.515	0.454	1.014	0.718-1.430	0.939
>50	61.199 (4.064)	53.234-69.165				
**Gender**						
Female	54.566 (4.467)	45.810-63.321	0.210	0.718	0.484-1.064	0.099
Male	57.363 (3.886)	49.747-64.978				

Abbreviations: *CI* Confidence interval, *Exp(B)* Odds ratio, *SE* Standard error, *LNM* Lymph node metastasis;

* *P* < 0.05

To further investigated whether the overexpressed p-S6 was an independent prognostic index for NSCLC patients, Cox regression analysis was carried out. The results shown in Table 3 indicated that up-regulated p-S6 might be a poor prognostic marker for patients with NSCLC (*p =* 0.011), as are clinical stage (*p <* 0.001), LNM status (*p =* 0.015) and pathological grade (*p =* 0.013). Besides, age, gender, histological type had no significantly effect on the prognosis of patients with NSCLC.

## Discussion

In this study, we demonstrated that the level of p-S6 protein expression in NSCLC was significantly higher than that in Non-CLT. The percentage of high p-S6 expression was obviously increased in patients with LNM than that without LNM. Inhibition of p-S6 expression reduced the invasion and migration ability of NSCLC cells. Our analysis indicated that NSCLC patients with increased p-S6 expression had a lower rate of survival than that with low expression ones. Taking together, our results imply that p-S6 may play a significant part in the progression of NSCLC and aberrant expression of p-S6 might be a novel prognostic marker for NSCLC. Increasing evidences have shown that mTOR/S6K/S6 pathway plays a crucial role in p53-mediated tumor inhibition [27, 28]. P-S6, the key downstream effector of mTOR/S6K pathway, is involved in the occurrence and development of many cancers [9]. Its expression is ascendant in numerous tumors, such as RCCs, pancreatic cancer, and esophageal squamous cell carcinoma [18, 27, 29]. The phosphorylation of S6 can attenuate the autophagy induced by damage-regulated autophagy modulator 1 (DRAM1) and p-S6 is a requirement for AKT-driven malignant transformation of pancreatic islet β cells [28, 30]. Due to the oncogenic role of p-S6, descending its expression could potentially provide a clue to find new idea for the targeted treatment cancers. For example, the suppression of p-S6 can block the further inhibition of the therapeutic mTOR inhibitor everolimus on protein synthesis and proliferation of RCCs cells [18]. Knockdown of S6K1 gene can obviously decrease the expression of cyclin D, leading to the decline of survival ability of esophageal cancer cells [29].

Activating invasion and metastasis is one of biological capabilities of cancers [31]. Our study showed that NSCLC patients with LNM had higher p-S6 expression. Furthermore, the expression level of p-S6 in metastatic lymph node lesion was higher than that in matched primary lesion. Multivariate analysis indicated that the high p-S6 expression could be an independent predicted marker for LNM in patients with NSCLC. Ribosomal protein S6, as a famous effector of mTOR signal pathway, mostly involved in the regulation of cell size and proliferation [9]. However, there is rarely report about the effect of p-S6 on metastasis of NSCLC. To further clarify the metastatic ability of p-S6 in NSCLC, we inhibited p-S6 expression in A549 and SPC-A1 cell lines and found that down regulation of p-S6 weakened the migration and invasion ability of NSCLC cells. Taking together, these data suggest that p-S6 might play a major part in promoting invasion and metastasis of NSCLC. Similar to our discoveries, previous studies have reported that overexpressed p-S6 is positively related to LNM in RCCs, colorectal cancer, and epithelial ovarian cancer [18, 32, 33]. On the other hand, inhibition of p-S6 expression or S6 gene knockdown can significantly suppress the cell invasion and migration of several kinds of human cancers, such as esophageal cancer and colorectal cancer cells [29, 34]. All the mentioned above indicate p-S6 positively affects cell invasion and migration. Despite the number of studies describing the aberrant expression of p-S6 in tumors activating invasion and metastasis, the concrete biological mechanism is still unclear. One study report that the phosphorylation defect of S6 inhibits the phosphorylation of paxillin, a focal adhesion protein, leading to inhibit the formation of local adhesion [29]. In this current study, we have little knowledge of how the aberrant expression of p-S6 involved in the LNM of NSCLC, further explorations for the intrinsic mechanism are required in the future.

Our results indicated that the OS rate of NSCLC patients with highly expressed p-S6 was obviously lower than that of those with low level of p-S6. Multivariate analysis showed that high expressed p-S6 was an independently prognostic indicator in NSCLC patients, which seem to favor the oncogenic role of p-S6. Previous studies on RCCs, gastric cancer, and glioblastomas also identified p-S6 as a novel poor prognosis biomarker [16–18]. Up to now, there are many inhibitors that can prevent S6 phosphorylation. Studies have reported that inhibition of p-S6 can significantly reduce tumor growth, which is important for effective response to treatment of triple negative breast cancer [35, 36]. In addition, mTOR inhibitor everolimus can effectively inhibit the level of p-S6, thereby reversing the resistance of *HER2*-mutant cancers to neratinib and exerting anti-tumor effects [37]. These further suggest that p-S6 may be a powerful biomarker in tumors and may provide novel strategy in targeted therapy of NSCLC.

## Conclusions

In summary, a significant overexpression of p-S6 was found in NSCLC. Inhibition of p-S6 expression could weakened the migration and invasion ability of NSCLC cells and aberrant expression level of p-S6 might be an independent predictor for LNM of NSCLC patients. In addition, overexpressed p-S6 may be a novel poor prognostic biomarker for NSCLC patients.

## Supplementary Information

**Additional file 1.**

**Additional file 2.**

## Data Availability

All data generated or analyzed during this study are included in this published article (and its supplementary information files). The datasets generated and analysed during the current study are available in the figshare repository, 10.6084/m9.figshare.16901161.v4.

## Abbreviations

NSCLC: Non-small cell lung cancer
ADC: Adenocarcinoma
SCC: Squamous cell carcinoma
LNM: Lymph node metastasis
AKT: The protein kinase B
mTOR: Mammalian target of rapamycin
S6: Ribosomal protein S6
S6K: 70 kDa S6 Kinases
P-S6: Phosphorylated S6
RCCs: Renal cell carcinomas
TMAs: Tissue microarrays
Non-CLT: Non-cancerous lung tissues
