# Supplementary material for: Overexpressed p-S6 associates with lymph node metastasis and predicts poor prognosis in non-small cell lung cancer
Source: BMC Cancer. 2022 May 20;22:564. doi: 10.1186/s12885-022-09664-4 (PMC9123697; doi:10.1186/s12885-022-09664-4)
Supplement: Supplementary file 1 — Additional file 1. [file 12885_2022_9664_MOESM1_ESM.pdf]

Supplementary Table. Clinicopathological features of patients with NSCLC and non-cancerous lung tissues

**Patients characteristics**	No. of patients (%)
**NSCLC**	
**Age(years)**	
≤50	95(27.1)
＞50	255(72.9)
**Gender**	
Male	266(76.0)
Female	84(24.0)
**Clinical stages**	
Stage Ⅰ	76(21.7)
Stage Ⅱ	75(21.4)
Stage Ⅲ	199(56.9)
**Lymph node status**	
N0	140(40.0)
N1/N2/N3	210(60.0)
**Histological type**	
SCC	154(44.0)
ADC	196(56.0)
**Pathological grade**	
Well	6(1.7)
Moderate	146(41.7)
Poor	198(56.6)
**Non-cancerous lung tissues**	
**Age(years)**	
≤50	22(41.5)
＞50	31(58.5)
**Gender**	
Male	27(50.9)
Female	26(49.1)
