# Supplementary material for: Overexpressed p-S6 associates with lymph node metastasis and predicts poor prognosis in non-small cell lung cancer
Source: BMC Cancer. 2022 May 20;22:564. doi: 10.1186/s12885-022-09664-4 (PMC9123697; doi:10.1186/s12885-022-09664-4)

Supplementary Figure 1. Quantitative analysis of p-S6 expression in lung SCC and ADC compared to Non-CLT

The level of p-S6 expression was significantly higher in lung SCC and ADC (p = 0.031, p < 0.001, respectively).

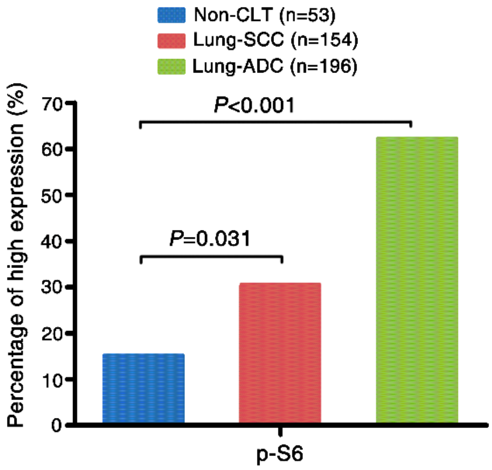

Supplement: Supplementary file 2 — Additional file 2. [file 12885_2022_9664_MOESM2_ESM.zip › Supplementary Figure 1.pdf]
