# Supplementary material for: Overexpressed p-S6 associates with lymph node metastasis and predicts poor prognosis in non-small cell lung cancer
Source: BMC Cancer. 2022 May 20;22:564. doi: 10.1186/s12885-022-09664-4 (PMC9123697; doi:10.1186/s12885-022-09664-4)

Supplementary Figure 2 Original blots for Fig 3. Considering the need to incubate different antibodies, the corresponding parts of the membrane were cut before incubation. After incubation and development of p-S6 (ser235/236) antibody, the relevant bands were stripped and then used to incubate S6 antibody. (A) and (B) are from different gels. (A) Full length membranes and original blots for A549 cells. (B) Full length membranes and original blots for SPC-A1 cells.

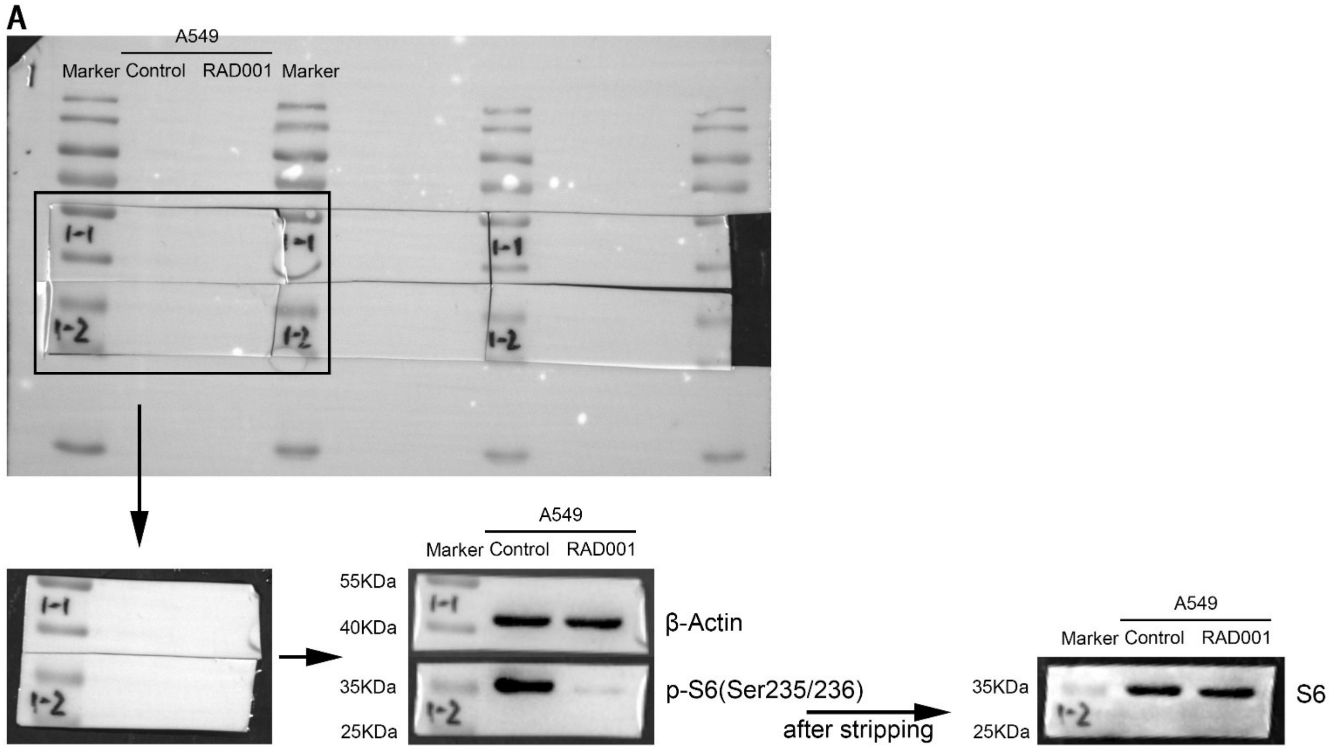

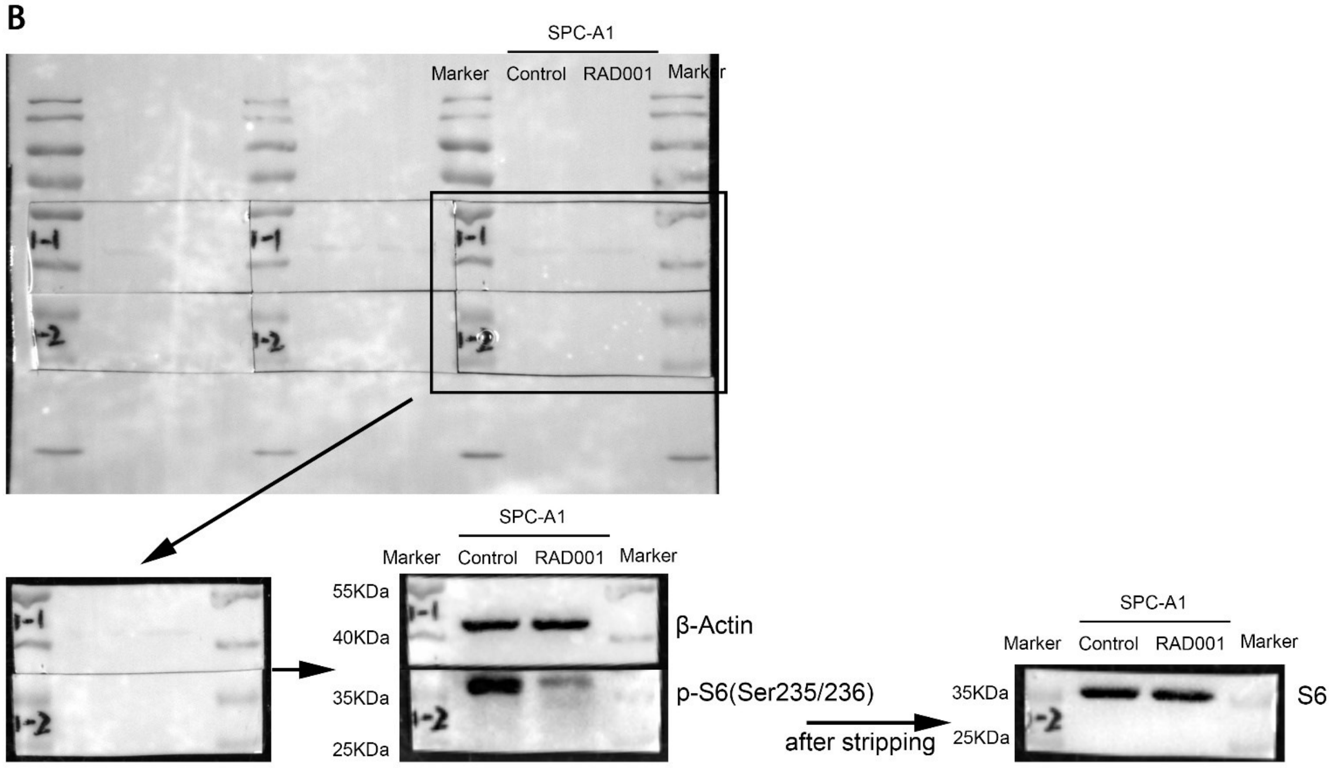

Supplement: Supplementary file 2 — Additional file 2. [file 12885_2022_9664_MOESM2_ESM.zip › Supplementary Figure 2.pdf]
